# Dosimetric benefits of IMRT and VMAT in the treatment of middle thoracic esophageal cancer: is the conformal radiotherapy still an alternative option?

**DOI:** 10.1120/jacmp.v15i3.4641

**Published:** 2014-05-08

**Authors:** Zhiqin Wu, Congying Xie, Meilong Hu, Ce Han, Jinling Yi, Yongqiang Zhou, Huawei Yan, Xiance Jin

**Affiliations:** ^1^ The Department of radiotherapy and Chemotherapy the 1st Affiliated Hospital of Wenzhou Medical College Wenzhou China

**Keywords:** middle thoracic esophageal cancer, conformal radiotherapy, intensity‐modulated radiotherapy, volumetric‐modulated arc therapy

## Abstract

The purpose of this study is to investigate the dosimetric differences among conformal radiotherapy (CRT), intensity‐modulated radiotherapy (IMRT), and volumetric‐modulated radiotherapy (VMAT) in the treatment of middle thoracic esophageal cancer, and determine the most appropriate treatment modality. IMRT and one‐arc VMAT plans were generated for eight middle thoracic esophageal cancer patients treated previous with CRT. The planning target volume (PTV) coverage and protections on organs at risk of three planning schemes were compared. All plans have sufficient PTV coverage and no significant differences were observed, except for the conformity and homogeneity. The lung V5, V10, and V13 in CRT were 47.9% ± 6.1%, 36.5% ± 4.6%, and 33.2% ± 4.2%, respectively, which were greatly increased to 78.2% ± 13.7% (*p* < 0.01), 80.8% ± 14.9% (*p* < 0.01), 48.4% ± 8.2% (*p* = 0.05) in IMRT and 58.6% ± 10.5% (*p* = 0.03), 67.7% ± 14.0% (*p* < 0.01), and 53.0% ± 10.1% (*p* < 0.01) in VMAT, respectively. The lung V20 (*p* = 0.03) in VMAT and the V30 (*p* = 0.04) in IMRT were lower than those in CRT. Both IMRT and VMAT achieved a better protection on heart. However, the volumes of the healthy tissue outside of PTV irradiated by a low dose were higher for IMRT and VMAT. IMRT and VMAT also had a higher MU, optimization time, and delivery time compared to CRT. In conclusion, all CRT, IMRT, and VMAT plans are able to meet the prescription and there is no clear distinction on PTV coverage. IMRT and VMAT can only decrease the volume of lung and heart receiving a high dose, but at a cost of delivering low dose to more volume of lung and normal tissues. CRT is still a feasible option for middle thoracic esophageal cancer radiotherapy, especially for the cost‐effective consideration.

PACS numbers: 87.53.Kn, 87.55.x 87.55.D 87.55.dk

## I. INTRODUCTION

Due to its dose painting ability compared to 3D conformal radiation therapy (CRT), intensity‐modulated radiation therapy (IMRT) has been applied more and more in the treatment of esophageal cancer for the clinical benefits of sparing lung and spinal cord. The present experience in the application of IMRT showed acceptable dose homogeneity within the planning target volume (PTV) and reduced lung irradiation.^(1)^ The main drawbacks of IMRT despite its efficiency in dose conformity to tumor are increased treatment delivery time and monitor units (MU). The increased treatment time will increase the patients' discomfort. Increased MU causes relatively larger low‐dose volume on organs at risk (OAR) and normal tissues, which may lead to side effects, such as radiation pneumonitis,^(2)^ as well as inducing secondary cancer.^(3)^

Comparative dosimetric study between volumetric‐modulated arc therapy (VMAT) and IMRT in the treatment of distal esophageal cancer indicated that VMAT is capable of delivering plans with OAR sparing and PTV coverage similar to IMRT, while reducing the number of MUs required and overall treatment time. The reduced treatment time may come at the cost of delivering small doses of radiation to a larger volume of healthy tissue.^(4)^ Comparison between RapidArc (Varian Medical Systems, Palo Alto, CA) and fixed‐field IMRT in cervical esophageal cancer also demonstrated that RapidArc could achieve similar tumor coverage and OARs sparing with shorter delivery time and fewer MUs.^(5)^

Esophageal tumors below the carina, but not extending to the GE junction, are considered middle esophagus.^(6)^ Supraclavicaular nodes are usually not included in the radiation field for middle thoracic esophageal cancer, which is different from that of cervical esophageal cancer. Celiac nodes and low abdomen OARs, such as kidney and stomach, are usually not involved in the radiation field for middle thoracic esophageal cancer, which differs from that of distal esophageal cancer. Therefore, the target volume of middle esophageal cancer is much more regular than those of cervical and distal esophageal cancer. The purpose of this study is to study the dosimetric differences among CRT, IMRT, and VMAT in the treatment of middle esophageal cancer and determine the most appropriate radiotherapy treatment modality.

## II. MATERIALS AND METHODS

### A. Patients

Eight patients with middle thoracic esophageal cancer treated previously with CRT beams in our department were enrolled in this study. Patients were immobilized in supine position. CT simulation was acquired on a Philips Brilliant spiral CT (Philips Brilliant, Cleveland, OH) according to standard procedures with 3 mm slice spacing. The entire lungs were scanned for further plan evaluation. Each patient was replanned retrospectively via the Pinnacle treatment planning system (clinical version 9.2; Philips, Fitchburg, WI). Patient staging information according to the AJCC staging system (AJCC, 2002) and other relevant characteristics were summarized in Table 1.

**Table 1:** Patient staging and characteristics

*Patient*	*Staging*	*GTV (cm^3^)*	*CTV (cm^3^)*	*PTV (cm3)*
1	T4N0M1	15.3	108.5	170.1
2	T3N0M1	25.7	209.8	303.2
3	T4N1M0	25.6	246.1	356.3
4	T3N0M0	17.3	97.4	158.3
5	T3N1M0	19.4	246.3	351.3
6	T3N0M0	26.0	290.8	378.9
7	T4N0M1	18.4	235.6	322.3
8	T4N1M0	13.1	130.1	198.2

Gross tumor volume (GTV), clinical target volume (CTV), PTV, and nodes were contoured by a physician according to the RTOG 0436 protocol.^(7)^ The GTV included the gross tumor and involved nodes as defined by diagnostic CT, esophagogastroscopy, endoscopic ultrasound, and PET scan. The CTV was delineated with 3‐5 cm superior‐inferior margins and 1 cm lateral and anterior‐posterior margins with respect to the GTV. The PTV was delineated with a 0.5 cm margin from the CTV. The spinal cord, lung, and heart were contoured as OARs on each image by one dosimetrist consistently.

### B. Planning schemes

IMRT and one‐arc VMAT plans were created for each patient. Radiation dose of 60 Gy was adopted for these patients with radiotherapy as the primary treatment.^(8)^ The prescription dose of CRT treatment was 2Gy×18 fractions with a total dose of 36 Gy for the first antero‐posterior and postero–anterior (APPA) phase, and then followed by off‐cord CRT beams with a prescription of 2Gy×12 fractions. The prescription doses for the replanned IMRT and VMAT plans were 2Gy×30 fractions. Five equally spaced beams were set up for IMRT plans, which were 0°, 72°, 144°, 216°, and 288°. The direct machine parameter optimization (DMPO, a form of direct aperture optimization) algorithm was applied for IMRT optimization. The maximum number of iterations was limited to 150 and the maximum number of segments was 50. A minimum segment area of $4\;{\text{cm}}^{2}$ and a minimum segment MU of eight was applied. For VMAT plan optimization, a leaf motion of 0.46 cm/deg and a final arc space degree of 4 were employed. A start angle of 181° and a stop angle of 180° were applied for one arc plans using clockwise (CW) rotation direction, as described in our previous study.^(9)^ Final doses of all treatment schemes were calculated by collapsed cone convolution (CC convolution) algorithm.

All plans were optimized to reach clinically acceptable PTV coverage and OAR sparing. At least 95% of the PTV must be covered by 95% of the prescription dose. The maximum dose of spinal cord must be less than 45 Gy, and the percent volume of lung receiving 20 Gy (V20) and 30 Gy (V30) should be less than 30% and 20%, respectively. Identical objective settings were applied for IMRT and VMAT optimization, with a higher priority given to PTV coverage and spinal cord constraint compared to lung constraints.

### C. Plan evaluation and comparison

Numerous plan quality indices were calculated from dose‐volume histogram (DVH) data for evaluation and comparison, such as the maximum dose (Dmax), mean dose (Dmean), and the volume of PTV that is covered by 93% (V93) and 95% (V95) of the prescription dose. The average MU of two phases of CRT and the MUs of IMRT and VMAT were compared. The optimization time and delivery time of IMRT and VMAT were also compared to those of CRT.

Homogeneity index (HI) was evaluated as the difference between the dose to 1% (D1) and 99% (D99) of PTV divided by the prescription dose (Dp):^(10)^

(1) $$\text{HI}=\frac{D1-D99}{D_{P}}\times 100\%$$

Conformity index (CI)^(11)^ and conformation number (CN)^(12)^ were also calculated for PTV:

(2) $$\text{CI}=\frac{V_{T,\text{Pi}}}{V_{\text{Pi}}}$$

(3) $$\text{CN}=\frac{V_{T,\text{Pi}}}{V_{T}}\times \frac{V_{T,\text{Pi}}}{V_{\text{Pi}}}$$

where $V_{T,Pi}$ is the volume of target that is covered by the prescription dose, $V_{T}$ is the volume of target, and $V_{Pi}$ is the volume of the body that is covered by the prescription isodose. The maximum value of CI is 1, corresponding to a perfect coverage of PTV. The CN is the complementary information to compensate for the defects of target coverage and CI. CN can take values between 0 and 1, where an ideal dose distribution would have a CN value of 1.

Radiobiological ranking indices tumor control probability (TCP) and normal tissue complication probability (NTCP) were also calculated using the Niemierko model.^(13)^ The equivalent uniform dose (EUD) is obtained as:

(4) $$\text{EUD}={\left(\frac{1}{N}\sum_{1}^{N}D_{i}^{a}\right)}^{\frac{1}{a}}$$

where, *N* is the number of voxels in the structure of interest, $D_{i}$ is the dose in the ith voxel, and a is the tumor normal tissue‐specific parameter that describes the dose‐volume effect. Based on the equivalent uniform dose, the TCP can be calculated by

(5) $$\text{TCP}=\frac{1}{1+{\left[\frac{{\text{TCD}}_{50}}{\text{EUD}}\right]}^{4\gamma_{50}}}$$

where $TCD_{50}$ is the tumor dose required to produce 50% TCP, and $\gamma_{50}$ is the slope of dose response at 50% TCP. These tumor‐specific parameters were cited from the study of Okunieff et al.^(14)^

In the case of normal tissue, the NTCP is determined as

(6) $$\text{NTCP}=\frac{1}{1+{\left[\frac{{\text{TD}}_{50}}{\text{EUD}}\right]}^{4\gamma_{50}}}$$

where $TD_{50}$ is the dose at which the probability of complication becomes 50% in 5 years, and $\gamma_{50}$ is the slope of signoidal dose response curve of normal tissue at 50% complication probability. These tissue‐specific parameters are based on the Niemierko model.^(13)^ The NTCP of the spinal cord, heart, and lung were calculated for plan evaluation. Parameters applied in this study for TCP and NTCP calculation were summarized in Table 2.

**Table 2:** Parameters for TCP and NTCP calculation cited from Niemierko and Goiten^(13)^ and Okunieff et al.^(14)^

	*Esophageal*	*Brainstem*	*Spinal Cord*	*Lung*	*Heart*
TCD50 (Gy)	49.09				
Slope50 (%/Gy)	4.14				
γ50 (%/%)	2.16	3	3	2	3
α	−13	7	13	1	3
TD50/5 (Gy)		65	66.5	24.5	48

The mean dose (Dmean) and maximum dose (Dmax) for OARs, the volume of heart receiving 25, 30, and 50 Gy (V25, V30, V50), and the volume of lung receiving 5, 10, 13, 20, 30 Gy (V5, V10, V13, V20, V30) were calculated and compared. The volume of normal tissue outside of the PTV receiving 5, 10, and 15 Gy (V5, V10, V15) were also evaluated.

### D. Statistical analysis

Results were described as mean±standard deviation (SD). Comparisons among different treatment modalities were analyzed with one way ANOVA method. When an overall significant difference was observed, the post hoc Turkey's test was used to determine which pair‐wise comparisons differed. All statistical analysis was conducted with SPSS 17.0 software. Difference was considered statistically significant when p<0.05.

## III. RESULTS

**Figure 1:**
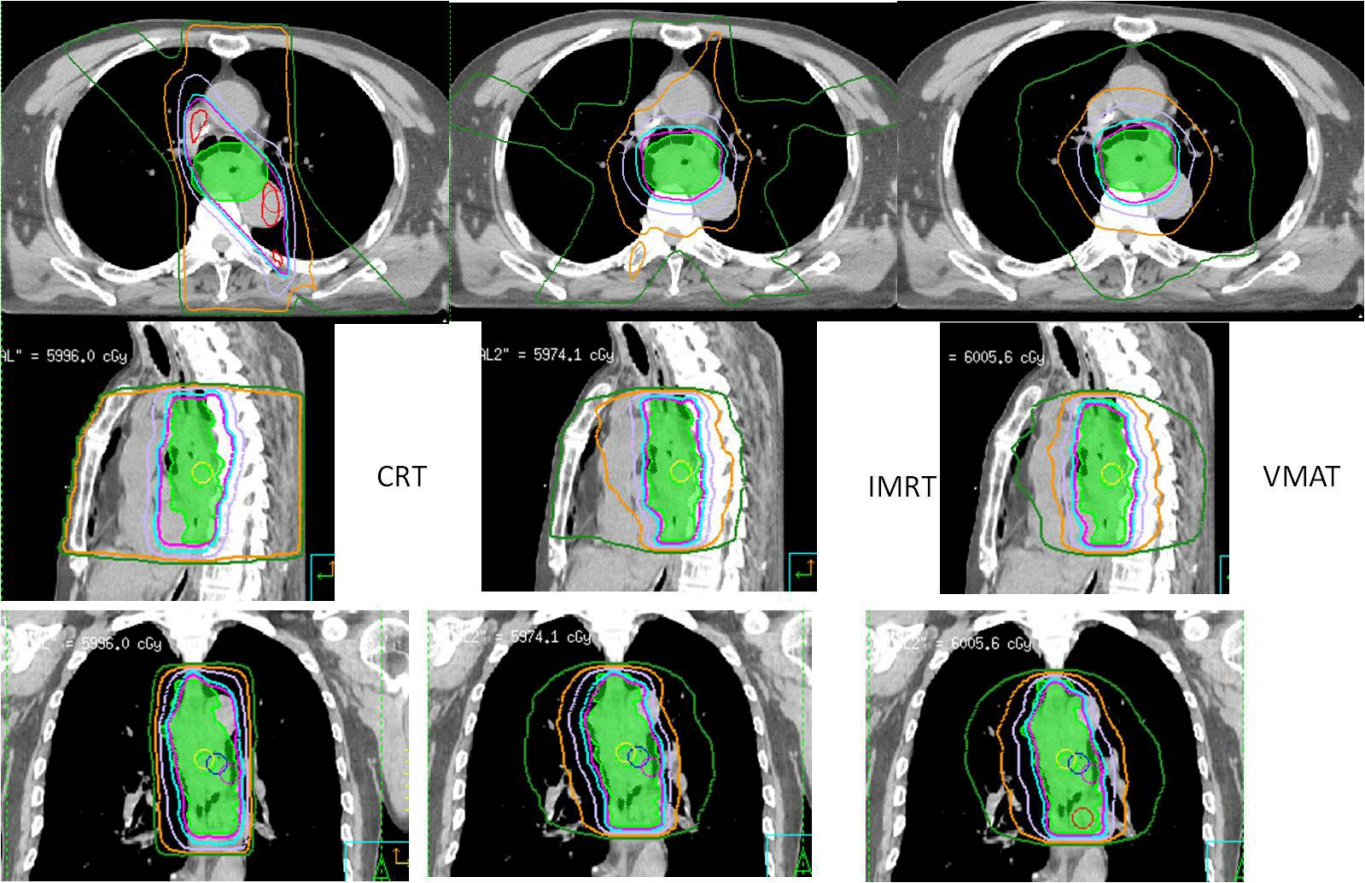
Dosimetric comparison among CRT, IMRT, and VMAT for one middle thoracic esophageal cancer patient.

**Figure 2:**
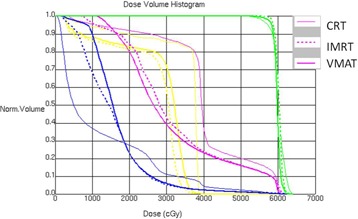
DVH comparison for one middle thoracic esophageal cancer patient.

One typical dose distribution comparison among CRT, IMRT, and VMAT plans for one middle thoracic esophageal cancer patient are shown in Fig. 1. The DVH comparison is shown in Fig. 2. For this specific patient, the CRT had the better lung sparing in the low‐dose area (<18Gy) and a higher dose in high‐dose area (>18Gy) to the IMRT or VMAT plans. Furthermore, compared to the IMRT and VMAT plans, the CRT had similar target coverage and a slight higher spinal cord dose.

Table 3 lists the target coverage comparison among three planning modalities. No significant difference on the Dmax, Dmean, EUD, and TCP were observed for PTV. Target coverage presented by V95 was improved by IMRT compared to CRT, but without significance (p=0.05). The CI, CN, and HI of IMRT and VMAT were 0.97±0.10,0.78±0.06,5.6±0.4, and 0.83±0.10,0.82±0.07, and 6.2±1.8, respectively, which were greatly improved (p≤0.01) compared to those of CRT plans with 0.47±0.20,0.49±0.21, and 10.6±3.9 for CI, CN, and HI, respectively. However, all plans meet the target prescription requirements.

**Table 3:** Target coverage comparison

				p‐value		
PTV	CRT	IMRT	VMAT	CRT vs. IMRT	CRT vs. VMAT	IMRT vs. VMAT
Dmax (cGy)	6397.5±175.4	6290.7±128.4	6184.4±109.0	0.38	0.02	0.38
Dmean (cGy)	6056.7±75.3	5985.9±63.4	6018.1±81.5	0.22	0.7	0.81
EUD (cGy)	6029.6±56.8	5980.4±63.3	6010.3±79.9	0.46	0.94	0.8
TCP	0.974±0.004	0.970±0.006	0.972±0.006	0.48	0.92	0.85
V93	99.4±0.9	99.9±0.0	99.8±0.2	0.13	0.36	0.93
V95	98.8±1.2	99.9±0.1	99.5±0.4	0.05	0.43	0.65
CI	0.5±0.2	0.8±0.1	0.8±0.1	<0.01	<0.01	1.00
CN	0.5±0.2	0.8±0.1	0.8±0.1	<0.01	<0.01	1.00
HI	0.999±0.001	0.999±0.001	0.991±0.01	1.00	0.56	0.50

Table 4 lists the OAR protection comparison among three planning modalities. The V5 and V10 of IMRT and VMAT were 78.2±13.7(p<0.01),80.8±14.9(p<0.01), and 58.6±10.5(p=0.03),67.7±14.0(p<0.01), respectively, which were greatly increased compared to those of CRT. CRT also achieved a lower volume in V13 compared to IMRT (p=0.05) and VMAT (p≤0.01). The volume of lung receiving a higher dose (V20;p=0.03,V30;p=0.05) in VMAT was lower than those of CRT. IMRT achieved a lower value in V30(p=0.04), but no significant differences were observed for V20(p=0.30) compared to CRT. IMRT and VMAT achieved a better protection on heart compared to CRT, but only V30 showed significant differences, with

**Table 4:** OAR sparing comparison

				p‐value		
	*CRT*	*IMRT*	*VMAT*	*CRT vs. IMRT*	*CRT vs. VMAT*	*IMRT vs. VMAT*
*Cord*						
Dmax (cGy)	4021.9±197.9	3871.2±142.4	3499.7±1013.1	0.98	0.48	0.73
Dmean (cGy)	2935.0±353.9	2080.7±318.9	1891.6±701.4	0.02	0.003	0.89
EUD (cGy)	3686.9±63.7	2989.5±158.2	2697.6±845.8	0.12	0.013	0.76
NTCP $\left(\times 10^{-4}\right)$	61.3±9.1	10.8±4.4	10.8±9.4	<0.01	<0.01	1.00
*Lung*						
Dmean (cGy)	1244.6±134.1	1422.6±213.0	1467.1±198.05	0.26	0.11	0.97
NTCP	0.0054±0.0027	0.019±0.015	0.022±0.014	0.18	0.06	0.94
V5	47.9±6.1	78.2±13.7	80.8±14.9	<0.01	<0.01	1.00
V10	36.5±4.6	58.6±10.5	67.7±14.0	0.03	<0.01	0.38
V13	33.2±4.2	48.4±8.2	53.0±10.1	0.05	<0.01	0.67
V20	27.5±3.5	24.3±3.7	22.2±3.8	0.30	0.03	0.64
V30	13.2±3.3	8.6±3.3	8.8±3.3	0.04	0.05	1.00
*Heart*						
Dmean (cGy)	2942.5±993.0	2332.4±937.6	2264.4±873.6	0.56	0.48	1.00
EUD (cGy)	3773.8±694.1	3128.5±682.7	3087.6±616.9	0.22	0.18	1.00
NTCP	0.11±0.10	0.021±0.023	0.016±0.017	0.12	0.008	0.99
V25	63.2±23.0	48.4±23.2	40.1±18.5	0.51	0.15	0.86
V30	58.4±22.5	34.7±16.7	28.6±12.8	0.04	0.007	0.88
V50	14.5±8.0	8.7±5.9	8.9±5.6	0.30	0.33	0.88
*Body*						
V5	39.1±8.89.1±8.8	54.4±11.8	58.9±13.5	0.07	0.01	0.87
V10	32.7±7.5	38.2±9.7	43.7±11.5	0.71	0.17	0.71
V15	29.3±6.9	30.2±7.7	28.8±8.5	1.00	1.00	1.00

p=0.04 and p=0.007, respectively. The low dose (V5) delivered to the healthy tissue outside of PTV was increased by IMRT (p=0.07) and VMAT (p=0.01) compared to CRT.

The average MUs of CRT, IMRT, and VMAT were 292.3±15.0, 377.6±91.9 and 305.5±43.4, respectively. The optimization time for IMRT and VMAT were 2.4±0.8 and 14.5±3.7 minutes, respectively. The delivery time for CRT, IMRT, and VMAT were 1.1±0.02, 4.3±0.5, and 1.5±0.05 minutes, respectively, according to the MOSAIQ record and verify system (version 1.60Q3; IMPAC Medical Systems, Inc., Sunnyvale, CA).

## IV. DISCUSSION

Dosimetric differences of IMRT and VMAT plans in the treatment of middle thoracic esophageal cancer patients were investigated by comparing with CRT plans in this study. IMRT and VMAT plans improved the dose conformity and homogeneity, but no clear advantages on PTV coverage were observed compared to CRT. IMRT and VMAT plans decreased the volume of normal tissues irradiated by a high dose, but increased the volume of normal tissues irradiated by a low dose.

In this study, the PTV coverage of IMRT was not significantly improved compared to CRT for middle esophageal cancer, except for V95. This was different from the result of a previous study of Fenkell et al.,^(15)^ in which IMRT vs. 3D CRT in the treatment of the cervical esophageal cancer was compared. In that study, the median coverage of the PTV 50/56, and PTV 70 were all improved with IMRT. The difference could be from a relative simple target shape of middle esophageal cancer compared to cervical esophageal cancer. The advantage of IMRT with dose painting ability was more prominent for complex targets. For even more complicated target of head and neck cancer, numerous studies had confirmed the superiority of IMRT in target coverage compared to CRT.^(16)^ The dose conformity of IMRT and VMAT was improved for middle esophageal cancer compared to CRT. This was consistent with the study of Fenkell et al.^(15)^ The study of Vivekanandan et al.^(17)^ also confirmed the superiority of IMRT and VMAT in target dose conformity compared to CRT for esophageal cancer. The dose homogeneity of IMRT and VMAT plans was improved compared to those of CRT plans.

IMRT and VMAT decreased the maximum dose to spinal cord, but no significant difference was observed. This was consistent with the study of Vivekanandan et al.^(17)^ Moreover, the maximum spinal cord doses from all techniques were below the tolerance. The CRT showed the lowest mean lung dose, though no significant difference was observed. IMRT and VMAT plans irradiated more lung volume with a low dose compared to CRT. Similar conclusion had been reported that full course of IMRT plans produce more conformal high‐dose distributions to the PTV at the cost of low doses to more normal lung tissue.^(18)^ Similar to the study of Vivekanandan et al., the V30 of lung was greatly decreased with IMRT and VMAT compared to CRT. However, the decrease of V20 by IMRT was not statistically significant compared to CRT in our study. For the heart sparing, only V30 demonstrated a statistical significance for CRT with IMRT or VMAT. Compared to CRT, more volume of normal tissue was irradiated by IMRT and VMAT in this current study. The protection on heart and irradiation on normal tissue were consistent with the results presented in the study of Mayo et al.^(19)^

In our study, no difference on PTV coverage and OAR sparing between IMRT and VMAT was observed for middle esophageal cancer. Similar OAR sparing and PTV coverage of IMRT and VMAT in the treatment of distal esophageal cancer were also reported.^(4)^ However, Vivekanandan et al.^(17)^ reported that the single‐arc plan showed improvements in OAR sparing compared with IMRT plan, but it is inferior in terms of target coverage, while a double‐arc plan resulted in reduction of dose to OAR and healthy tissues with better target coverage. Yin et al.^(5)^ also concluded that RapidArc could achieve the similar tumor coverage as fixed‐field IMRT with effective OAR sparing in the treatment of cervical esophageal cancer. These differences could be caused by the various contour complexities of different part of esophageal cancer in different studies. Data from Guckenberger et al.^(20)^ indicated that the complexity of the target volume determined whether single arc VMAT was equivalent to IMRT. Bertelsen et al.^(21)^ found that a single arc was sufficient to achieve plan quality similar to IMRT, whereas another study stated that two or more arcs were required in treatment of the complex‐shaped target volumes.^(22)^ Martin et al.^(23)^ had also reported that using additional arcs could improve the PTV dose uniformity and homogeneity, and deliver a lower dose to lung at the cost of increased heart dose.

In this study, the MU of one‐arc VMAT was only slightly higher than CRT (*p* = 0.90), but the MU of IMRT was greatly increased (*p* = 0.02) compared to that of CRT. The optimization time of VMAT was greatly increased compared to IMRT (14.5±3.7 min vs.2.4±0.8 min). There is no need of optimization for CRT. Besides the easy beam setup, the final dose calculation of CRT will be less than 1 minute, which is much shorter compared to IMRT and VMAT. CRT also achieved the shortest delivery time compared to IMRT (p≤0.01) and VMAT (*p* = 0.04). VMAT achieved shorter delivery time compared to IMRT, as reported (*p* = 0.03).^(5)^

## V. CONCLUSIONS

In the treatment of middle esophageal cancer, all CRT, IMRT, and VMAT plans are able to meet the prescription, and there is no clear distinction on PTV coverage observed. IMRT and VMAT can only decrease the volume of lung and heart receiving a high dose, but at a cost of delivering low dose to more volume of lung and normal tissues. The CRT is still a feasible option for middle thoracic esophageal cancer radiotherapy, especially for the cost‐effective consideration.

## ACKNOWLEDGMENTS

The paper was supported by Wenzhou Science and Technology Bureau Funding (Y20120137) and the Scientific Research Foundation for the Returned Overseas Chinese Scholars (604090656/037).
